# WSe_2_ Monolayers Grown by Molecular Beam Epitaxy on hBN

**DOI:** 10.1021/acs.nanolett.5c04228

**Published:** 2025-12-07

**Authors:** Julia Kucharek, Mateusz Raczyński, Rafał Bożek, Anna Kaleta, Bogusława Kurowska, Marta Bilska, Sławomir Kret, Takashi Taniguchi, Kenji Watanabe, Piotr Kossacki, Mateusz Goryca, Wojciech Pacuski

**Affiliations:** † Institute of Experimental Physics, Faculty of Physics, 49605 University of Warsaw, Pasteura 5, 02-093 Warsaw, Poland; ‡ Institute of Physics, 86906 Polish Academy of Sciences, Aleja Lotników 32/46, 02-668 Warsaw, Poland; § Research Center for Materials Nanoarchitectonics, 52747 National Institute for Materials Science, 1-1 Namiki, Tsukuba 305-0044, Japan; ∥ Research Center for Electronic and Optical Materials, 52747 National Institute for Materials Science, 1-1 Namiki, Tsukuba 305-0044, Japan

**Keywords:** tungsten diselenide, molecular beam epitaxy, photoluminescence, atomic force microscopy, hexagonal boron nitride

## Abstract

A three-step process was developed for growing high-quality, optically uniform WSe_2_ monolayers by molecular beam epitaxy (MBE), taking advantage of the use of hexagonal boron nitride (hBN) as a substrate. The process was optimized to maximize the efficiency of photoluminescence and promote the formation of hexagonal WSe_2_ domains. Atomic force microscopy was employed to estimate the dispersion of the WSe_2_ hexagonal domains' orientation. Monolayer character of the film was identified using optical methods and verified with a high-resolution transmission electron microscopy cross-section. Temperature- and magnetic-field-dependent studies revealed the behavior of exciton complexes to be analogous to that of exfoliated counterparts. Direct growth on hBN, combined with a uniform optical response, proves that MBE-grown WSe_2_ is superior to mechanically exfoliated WSe_2_ in terms of the convenience of use and reproducibility. The provided results establish significant progress in the optical quality of epitaxially grown transition-metal dichalcogenide monolayers and the fabrication of large-scale functional devices.

## Graphical abstract

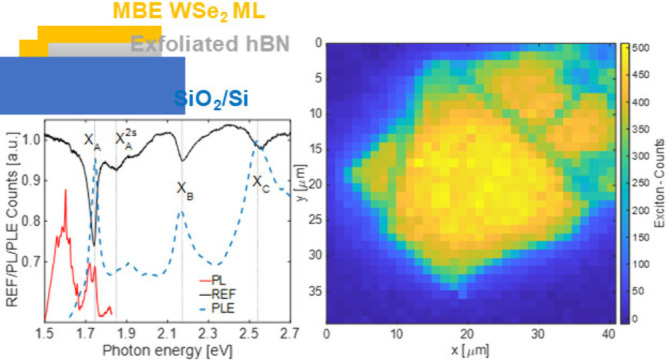

Since the beginning of the graphene era, two-dimensional (2D) materials have gained vast interest in the fields of electronics^1,2^ and optoelectronics.^3^ In particular, monolayers of transition-metal dichalcogenides (TMDs) from the MX_2_ group have attracted attention due to their direct band gap, the possibility of external dielectric screening tuning,^4^ and their very strong Coulomb interactions.^5^ WSe_2_, due to its positive conduction-band spin-splitting, allows the observation of multiparticle excitonic states, particularly dark/gray states.^6^

Although many successful bottom-up fabrications of WSe_2_ materials have been realized (Table 1), the optical properties of WSe_2_ are mostly studied on mechanically exfoliated layers encapsulated in hexagonal boron nitride (hBN),^7^ due to the efficiency of their optical response. So far, mechanical transfer and encapsulation in hBN were also needed for improvement of the optical quality of chemical vapor deposition (CVD)-grown WSe_2_ monolayers.^8^ In this work, the optical properties of as-grown WSe_2_ layers, revealed by low-temperature spectroscopy, are optimized for the first time.

**Table 1:** Overview of WSe_2_ Growth on Different Substrates by MBE, CVD, MOCVD, and AACVD Techniques with Information about the Estimated fwhm of the Excitonic Peak (If Present) and the Temperature of the Measurement^a^

technique	substrate	fwhm + temperature
MBE	sapphire	no PL^9^
MBE	mica	∼150 meV at RT^10^
MBE	AlN	no PL^11^
MBE	HOPG	no PL^12,13^
MBE	bilayer graphene	no PL^14^
MBE	epitaxial graphene	no PL^15^
MBE	ZrO	no PL^16^
MBE	Au	∼80 meV at RT^17^
MBE	GaP	no PL^18^
MBE	SrTiO_3_	no PL^19^
MBE	hBN	this work, 16.4 meV for X^0^, 23.8 meV for CX at 10 K
CVD	sapphire	∼200 meV at RT^20^
CVD	YIG	∼80 meV at 93 K^21^
CVD	SiO_2_	∼70 meV at RT;^22^ ∼50 meV at RT^23^
CVD	Si_3_N_4_	∼70 meV at RT^23^
CVD	epitaxial graphene	∼100 meV at RT^24^
CVD	fused silica	∼50 meV at RT^25^
CVD	Au	∼50 meV at RT^26^
CVD	hBN	∼40 meV for X^0^ at 80 K^27^
MOCVD	SiO_2_	∼50 meV at RT^28^
MOCVD	sapphire	no PL^29^
AACVD	sapphire	∼80 meV at RT^30^

a If PL was measured in more than one temperature, the data in the lowest one was placed in the table.

While exfoliated materials can be analyzed to find a particular working spot that has the desired optical properties, the same cannot be reliably said about its neighboring spots. In practice, the most desired materials are those that can be produced in a repeatable, schematized manner, resulting in large, uniform layers with predictable properties.

Addressing these demands, we present tungsten diselenide grown by molecular beam epitaxy (MBE) on hBN exhibiting high optical quality and uniformity over hundreds of square micrometers, limited only by the size of the available hBN substrate. Our WSe_2_ exhibits a photoluminescence (PL) signal with a set of emission lines originating from multiparticle excitonic states, with no need for upper hBN coverage. The MBE technique enables a well-defined, reproducible growth process directly on the hBN substrate. hBN flakes present many advantages as a substrate for TMD growth, e.g., a smooth surface without dangling bonds or uncompensated charges, which facilitates atom surface mobility. An atomic-terrace-free surface promotes continuous growth of the TMD film. Chemical and high-temperature resistance make hBN a convenient substrate that does not require complicated preparations before growth. 90 nm - thick SiO_2_ buffer on Si wafer and 10–20 nm of hBN^31^ effectively decrease the inhomogeneous broadening of the spectral lines,^32^ improving the optical quality of the material.

In previous works, we have shown successful results on the growth of bright MoSe_2_ monolayers on hBN.^33,34^ Notably, because WSe_2_ is a dark material, very sensitive to the deficiency of selenium, it requires precise adjustments of the growth parameters to achieve comparable results.

In this paper, we introduce MBE growth on hBN for high-quality, optically uniform WSe_2_ monolayers. The growth was realized in a three-step process, including pregrowth annealing to outgas the surface and flatten hBN flakes, a slow growth phase, and postgrowth annealing, facilitating tungsten atom rearrangement and hence improving the crystalline order of the layer.

In order to grow WSe_2_, an intrinsic (001) Si wafer with a 90 nm SiO_2_ layer was prepared in a chemical cleaning process, and then exfoliated hBN was transferred onto it, forming a complete substrate for growth. The scheme and optical microscopy image of the sample are depicted in Figure 1.

**Figure 1:**
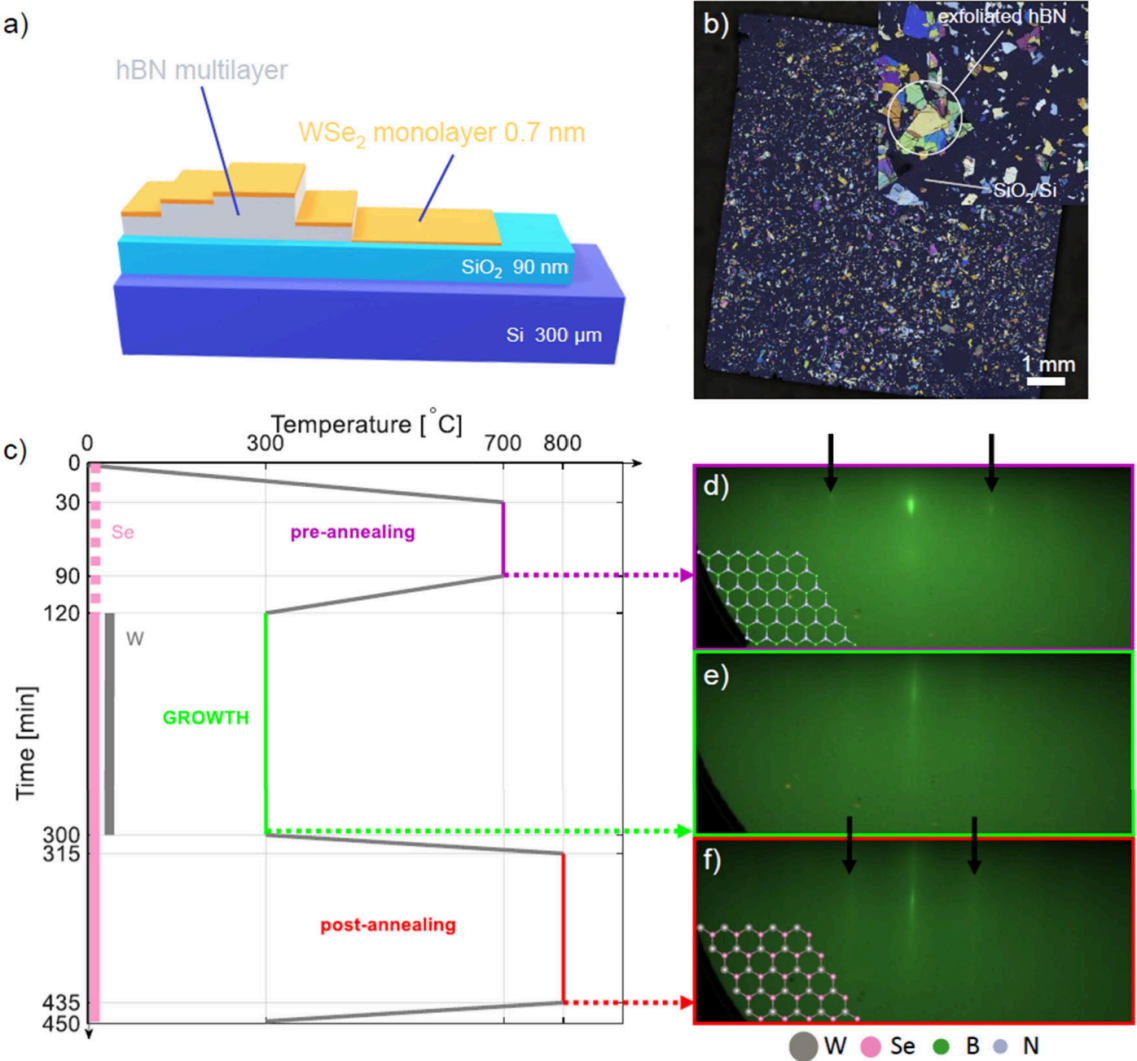
(a) Scheme of the sample. (b) Optical microscopy image of the sample. (c) Scheme of the growth process presenting substrate temperature evolution in time. (d–f) RHEED patterns of the annealed substrate before growth, the WSe_2_ sample after growth, and after final annealing, respectively.

Before growth, the substrate was annealed at 700 °C for 1 h, using MBE, to outgas the surface and relax the strain in exfoliated hBN flakes. Parallelly, the Se source was warming up to reach the growth target flux with the Se shutter open. Growth was performed at 300 °C for 2.5 and 3 h, respectively, for the two samples (UW2144 and UW2145) used for this paper. The tungsten e-beam source power set at 150 W corresponded to a 0.3 Å/h tungsten deposition rate. During growth, the selenium deposition rate is 8 × 10^3^ Å/h and changes to 4 × 10^4^ Å/h for the last step of the process. After growth, annealing was performed at 800 °C for 2 h. Finished WSe_2_ samples were removed from the growth chamber right after reaching the growth temperature for the second time to avoid selenium condensation on the surface. More details about the growth process can be found in Figure S6.

Reflection high-energy electron diffraction (RHEED) was used to monitor the growth in situ. In Figure 1d–f, each step of the growth is documented with RHEED images. After pregrowth annealing, hBN lines appear. During the following slow growth phase, only the WSe_2_ lines are visible. Finally, after postgrowth annealing, the lines are sharper and brighter, which suggests higher crystalline order. WSe_2_ strikes align with those of hBN, indicating epitaxial orientation of TMD domains with regard to the substrate. Assuming the initial hBN lattice constant to be 2.50 Å,^35^ one can estimate the WSe_2_ lattice constant to be 3.24 Å, which is less than 3.282 Å measured in ref 36 but close to the relaxed monolayer with an *a* = *b* lattice constant equal 3.247 Å claimed in ref 37 theoretical calculations.

For structural investigations of the WSe_2_ single-crystal film, we have used ambient atomic force microscopy (AFM). Atomic resolution images were acquired in ultrahigh vacuum using contact-mode AFM (UHVAFM). Figure 2 shows partially coalesced WSe_2_ flakes grown by MBE on the hBN substrate. The upper row displays pictures in three magnifications. The first one (Figure 2a) shows the general coverage of the surface of hBN to be 55%. Moreover, only 16% of the monolayer is covered with a second layer or more. The average roughness of the WSe_2_ surface in part a is 184 pm. Although the coverage of hBN with WSe_2_ is not 100%, keeping it at the level of 55% allows one to avoid second-layer formation and maximizes the optical response quality (see the comparison in Figure S6). Exfoliated hBN is an atomically smooth material supporting atom rearrangement that leads to the formation of many small TMD flakes. This effect was also shown in papers of the Redwing group, where, after the substrate was changed from sapphire to hBN, the WSe_2_ flake size changed from micrometers^20^ to ∼300 nm.^27^

**Figure 2:**
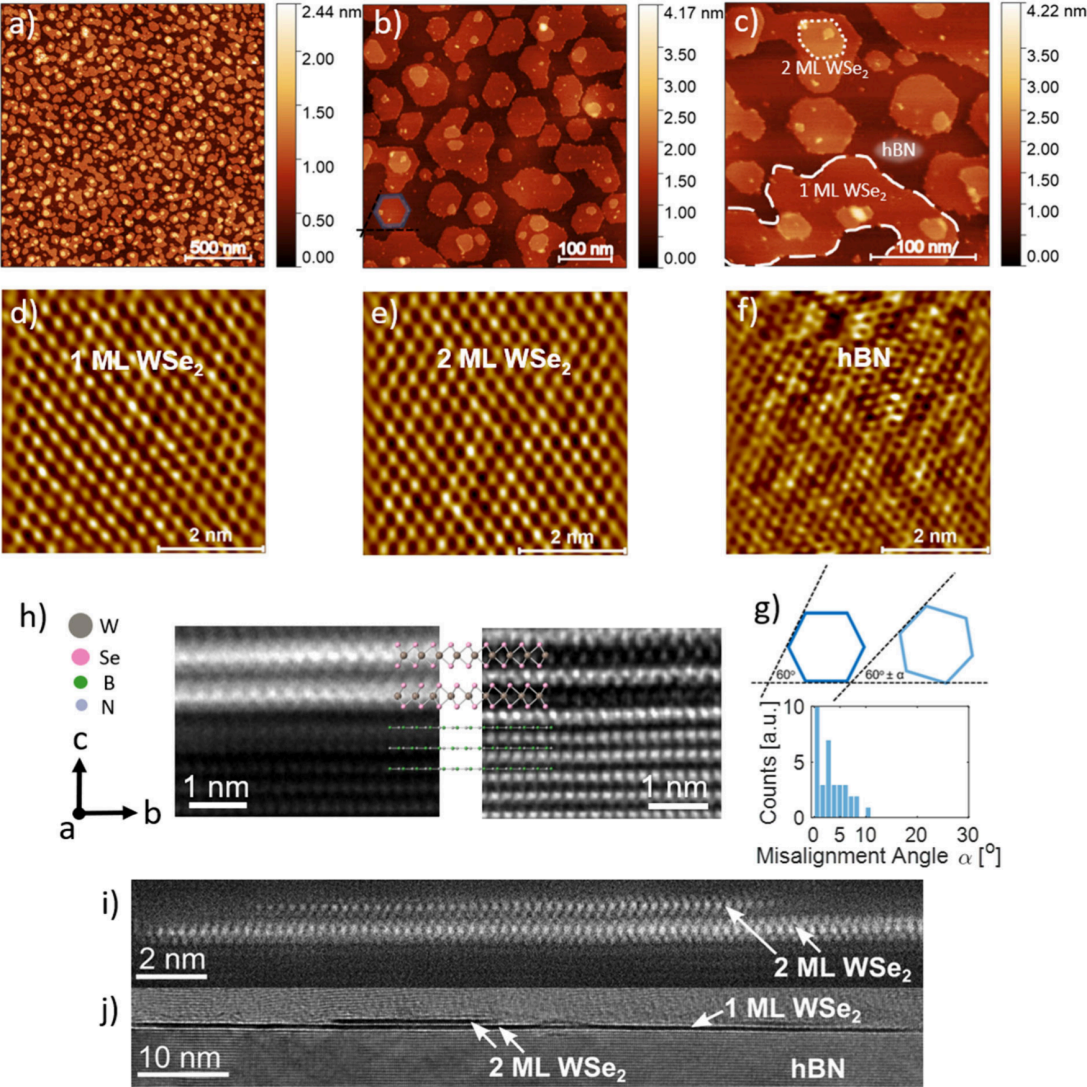
(a–c) AFM images of the UW2144 sample in three different magnifications. In part c, the monolayer, bilayer, and uncovered hBN areas are marked. (d–f) HRAFM images of the UW2144 sample surface. (g) Scheme showing the flakes’ misalignment angle α and histogram based on part b. TEM images of a WSe_2_ cross section: (h) Model of the WSe_2_ atomic structure superimposed on dark-field (HAADF-STEM, left) and bright-field (HR-TEM, right) images. (i) STEM-HAADF image showing two monolayers (MLs) of WSe_2_. The bright contrast corresponds to atomic columns. (j) HR-TEM image providing a wider view of the grown structure, showing areas with one or two MLs (dark region).

The tendency of WSe_2_ to form hexagonal flakes is well demonstrated in Figure 2b, proving that both W and Se edges in our material are equal and well-formed. In places where WSe_2_ forms bigger, coalesced flakes, one can also find 120° angles between edges. For each flake with at least one defined corner and straight edge, the average misalignment angle α of the flakes is 3.4°, as marked in Figure 2g.

WSe_2_ nucleation centers appear on both hBN (between the WSe_2_ flakes) and the already-grown WSe_2_ flakes (Figure 2c). There is no strong trend; nucleation centers are dispersed equally throughout the area. Exemplary zones of monolayer and bilayer of WSe_2_ and uncovered hBN are marked in Figure 2c. The bottom row of Figure 2 d-f shows UHVAFM images of a WSe_2_ monolayer, a WSe_2_ bilayer, and a multilayer of hBN, subsequently. Lattice constants of the materials were calculated as an average of 10 units measured along the three apical symmetry axes going through the hexagon corners. Estimations of the WSe_2_ and hBN lattice constants *a* and *b* are 3.51 and 2.71 Å for WSe_2_ (both monolayer and bilayer) and hBN, respectively. There is a noticeable difference in the lattice constant values between the RHEED and AFM measurements. To obtain high-quality images through AFM, the acquisition was slow and therefore susceptible to error due to the drift velocity. Thereupon, the values of the AFM lattice constants are overestimated.

Further characterization by TEM allows for assessment of the stacking regime of the grown material. Cross sections of the WSe_2_ monolayer and bilayer regions are shown in Figure 2h–j. Panel h presents the 2H stacking structure of two subsequent WSe_2_ layers. The atomic resolution inset illustrates the corresponding atomic arrangement. Scanning transmission electron microscopy in high-angle annular dark-field (STEM-HAADF) mode, which is sensitive to the atomic number (Z-contrast), directly indicates the location of atom columns and resolves W and Se atoms, as shown in Figure 2i. Panel j depicts a ∼100-nm-long WSe_2_ monolayer grown on a well-ordered hBN crystal, with a region of approximately 20 nm showing the presence of a second WSe_2_ layer. Although the WSe_2_ crystal does not form a complete monolayer, the film remains continuous over several hundred nanometers. The high-resolution TEM (HR-TEM) image in Figure 2j clearly reveals an exfoliated hBN structure. TEM images confirm van der Waals growth of both the WSe_2_ layer on hBN and the WSe_2_ bilayers. Furthermore, TEM data enable determination of the WSe_2_ lattice parameters, measured to be *a* = *b* = 3.3 Å and *c* = 13.1 Å. These values are in excellent agreement with the theoretical WSe_2_ structure and closely match RHEED estimations (∼1.5% variation), supporting the statement that UHVAFM values are overestimated.

To show MBE-grown WSe_2_ optical response characteristics, micro-PL, microphotoluminescence excitation (μ-PLE), and microreflectance (μ-REF) were studied. In Figure 3a, the PL spectrum (red line) shows a wide range of sharp lines characteristic of the WSe_2_ monolayer. Neutral (X^0^) and charged excitons (CX) at 1.745 and 1.72 eV, respectively, are marked. The black line, referring to the reflectance spectrum, shows exciton A absorption at 1.75 eV and excitons B and C located at 2.17 and 2.54 eV. The absorption minimum on 1.85 eV corresponds to the exciton A 2s state. The dashed (blue) line shows the PLE measurement, confirming the appearance of the positions of the A, A^2s^, B, and C excitons. Zero Stokes shift between PL and REF is confirmed by low-temperature (Figure 3a) and temperature-dependent (Figure 3e) studies.

**Figure 3:**
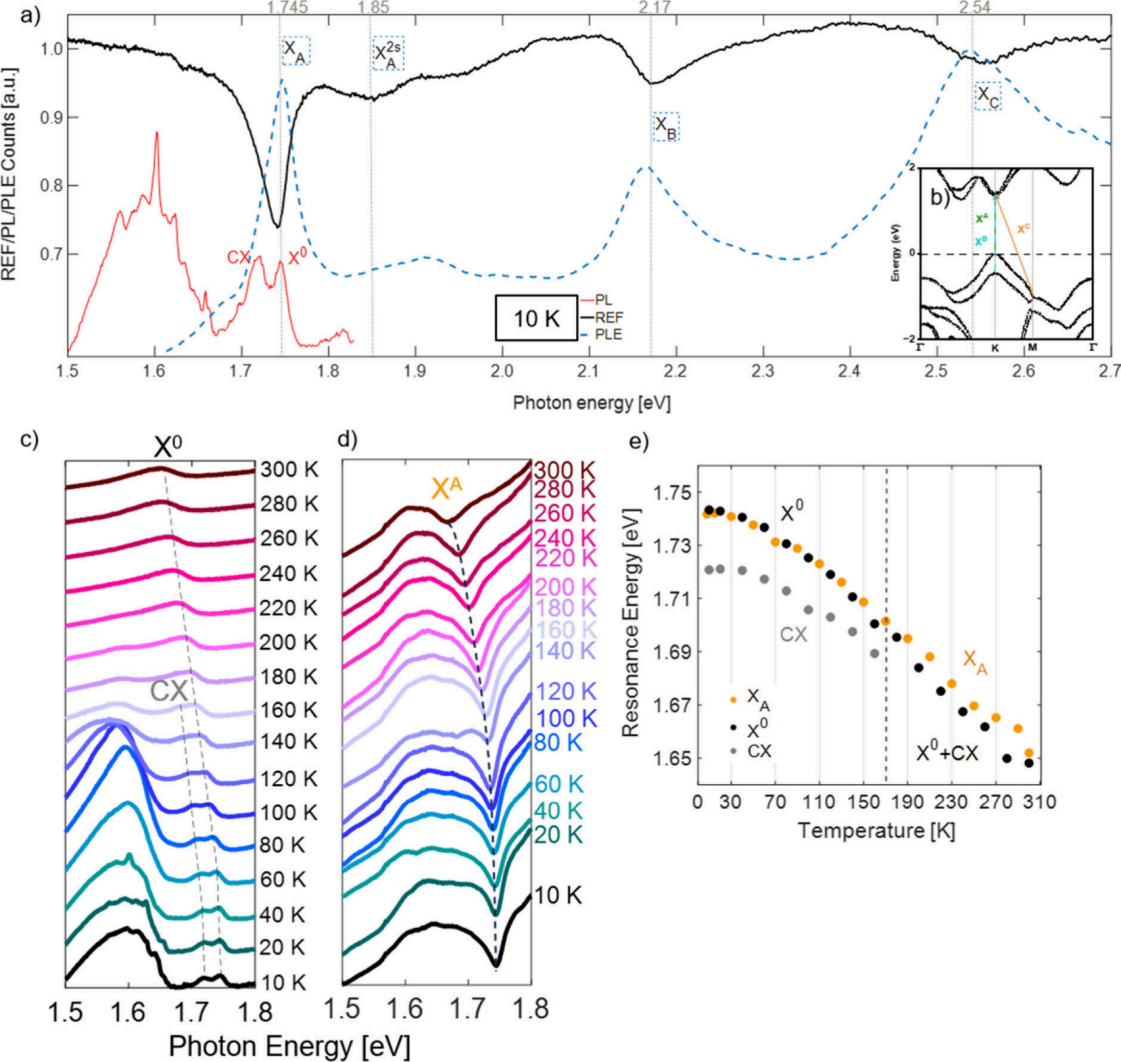
(a) Exemplary PL, reflectance, and PLE spectra of WSe_2_ measured in 10 K. (b) Exciton A, B, and C transitions in the WSe_2_ band structure.^38^ Band structure reproduced from ref 39. Available under a CC-BY license. Copyright 2018 Liu et al. (c) Neutral and charged exciton PL energy temperature dependence. (d) Exciton A absorption temperature dependence. (e) Comparison of the exciton A absorption energy dependence with neutral and charged exciton PL energy. A 532 nm laser with approximately 200 μW/μm^2^ power density was used.

For both PL and REF, we observe the temperature-dependent red shift and broadening of the lines. Analogous to the exfoliated counterpart, MBE-grown WSe_2_ reveals more intense emission from lines in the 1.6 eV band at temperatures between 10 and 210 K (Figure S4a), and this trend reverses for temperatures higher than 210 K. This is in contrast to exfoliated materials, where the integrated PL intensity is increasing monotonously with temperature.

The X^0^ energy (or exciton A) changes with temperature from 1.745 eV (at 10 K) to 1.649 eV (at 300 K) and reveals overlapping results with those published by Aurora et al.^38^ Comparisons of both are shown in Figure S3. After closer investigations of the X^0^ and CX intensity changes with temperature (Figure S4 and Table S1), it is clearly seen that thermal activation between the dark and bright states with an activation energy of ∼18 meV is observed. The same cannot be said about the 1.6 eV band, where the intensity increases up to 80 K and then drops. At an energy of ∼1.4 eV, another band appears (Figure S4a–c); its behavior is similar to that described above for the 1.6 eV band.

To reveal the exceptional optical homogeneity of the material, we show spatially resolved PL measurements (Figure 4a), where the intensity of the PL signal of X^0^ is displayed. The PL map precisely mirrors the measured hBN flake presented in Figure 4b. It is worth mentioning that, in Figure 4a, the PL intensity changes with respect to the hBN thickness. To clarify this effect, the small inset in part c with the cross-section of the studied flake is placed. The PL signal is similar and uniform on the surface of the ∼10-nm-thick hBN flake. The map is followed by a statistical analysis of the PL spectra collected on the hBN flake. All statistics exclude spectra from the outside of the flake but take into account all of the spots where hBN is inhomogeneous (e.g., wrinkles, edges, cracks, or folds). By fitting a Gaussian function (due to significant inhomogeneous broadening), we received neutral and charged exciton resonance energies, full widths at half-maximum (fwhm’s), and normalized exciton energies.

**Figure 4:**
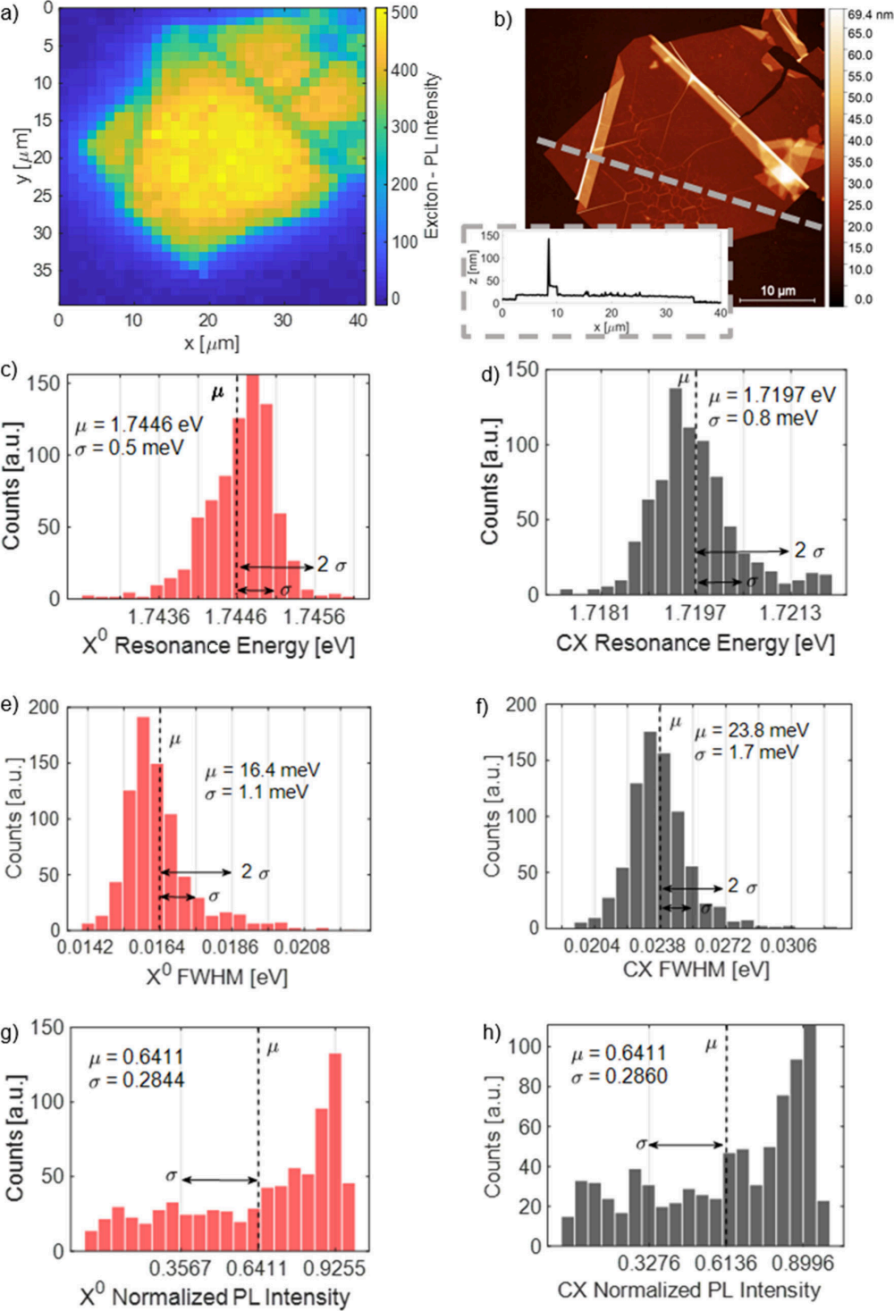
μ-PL map and statistics of WSe_2_ grown on hBN at low temperature (10 K): (a) Neutral exciton PL intensity map. (b) hBN flake mapped in part a. Histograms of neutral and charged exciton fitted parameters. The left column is dedicated to the neutral exciton, and the right column is dedicated to the charged exciton. (c and d) Resonance energy positions. (e and f) Full widths at half-maximum. (g and h) Normalized PL intensities. On each histogram, the average value and standard deviation are marked.

The average positions of X^0^ (μX^0^) = 1.7446 eV and CX (μCX) = 1.7197 eV are in agreement with the values published for exfoliated materials encapsulated in hBN measured at low-temperature PL.^40–42^ The standard deviation of the position is higher for the charged exciton than for the neutral one (0.5 and 0.8 meV, respectively). Analogically, the fwhm of the charged exciton reaches higher values than those for the neutral exciton, 1.1 and 1.7 meV, respectively. We can attribute this to the fact that the X^0^ resonance is composed of a single emission line, while CX consists of two emission lines: spin-singlet and spin-triplet trion.^43^ However, the average line widths for X^0^ and CX are notable, we can find lines as narrow as 10 meV for X^0^ and 15 meV for CX. They are still wider than near-homogeneous exfoliated (hBN-encapsulated) WSe_2_ FWHMs of 2 meV in refs 7 and 44, ∼4 meV in ref 32, or <5 meV in ref 45. First of all, we can attribute relatively large inhomogeneous broadening in MBE-grown WSe_2_ to the edge defect density due to the significant ratio of the WSe_2_ flakes’ edges to the WSe_2_ layer surface. The second largest factor is the substrate roughness.

To learn more about emission lines between 1.500 and 1.675 eV, parts a–c of Figures 4 and 5 present spatially resolved data of WSe_2_ grown on hBN. In part b, there is a map of the PL intensity integrated over energies from 1.500 to 1.675 eV, revealing a homogeneous answer varying from 600 to 900 counts depending on the particular spot on the flake from Figures 4b and 5b. Looking directly at the cross section of the map (Figure 5c), in the region of interest, from point to point, different sets of lines are visible. Because some lines repeat in a few spots, e.g., lines close to 1.60 eV or at 1.62 eV (marked with dashed lines), to verify their origin, we performed a temperature-dependent and power-dependent study (Figures S4 and S5). The measurements in a function of the temperature show that sharp lines like the one at 1.62 eV last to a maximum of 50 K, which suggests that they are not associated with phonon sidebands of momentum dark states.

**Figure 5:**
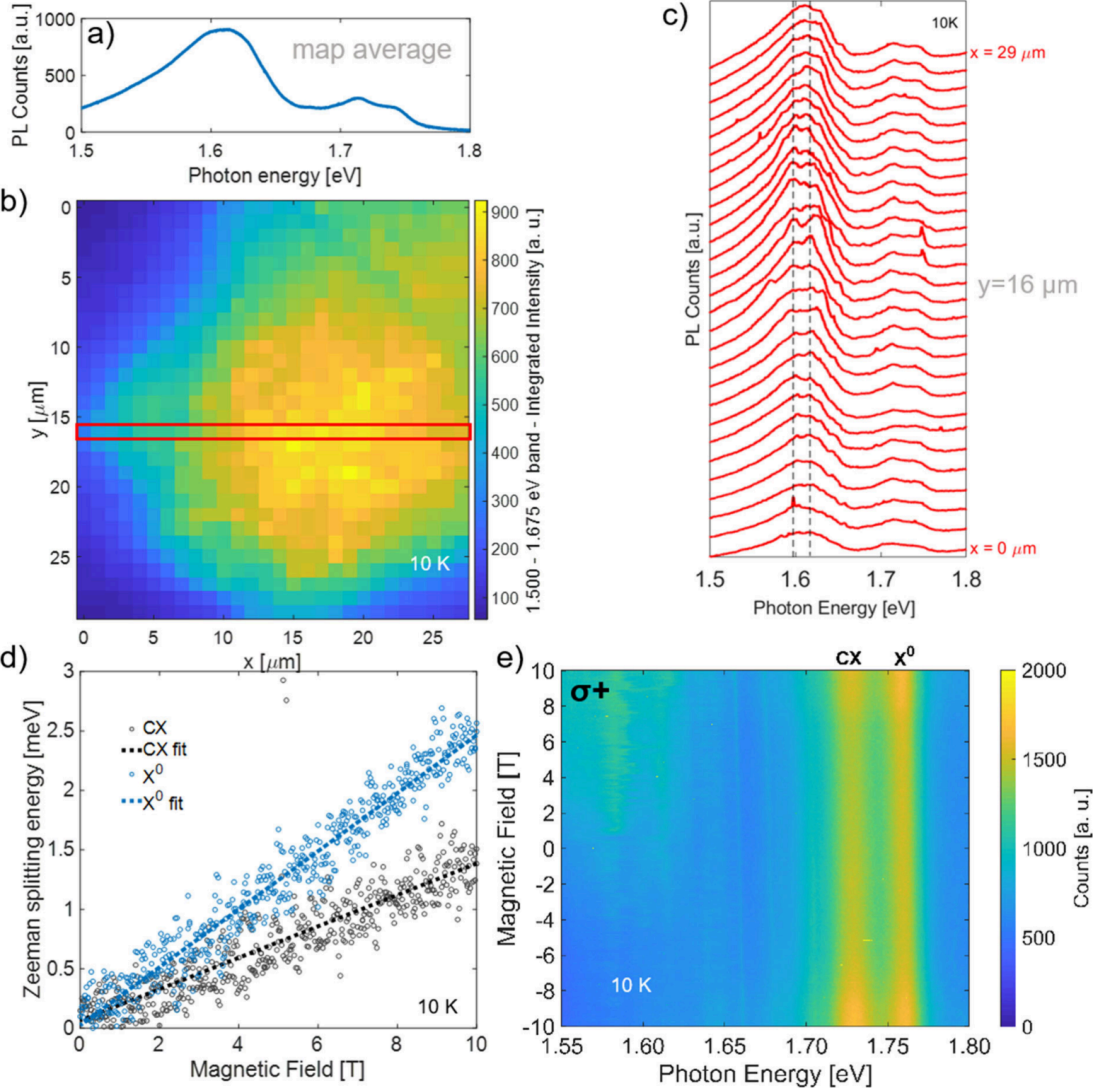
(a) PL spectrum calculated as an average over all points of the map in part b. (b) PL map illustrating the integrated intensity of the 1.500–1.675 eV band, collected on hBN flakes. (c) Evolution of the spectra through the chosen line on the map in part b (red rectangle) with two guiding eye lines. (d) σ+ polarization in the detection of PL as a function of the magnetic field. (e) Zeeman splitting energy of neutral and charged excitons with *g* factors of −4.4 for X^0^ and −2.4 for CX. Measurements were performed at low temperature (10 K) using 532 nm (a–c) and 635 nm (d and e) lasers for excitation.

In excitation power-dependent data, one can find lines from the region between 1.580 and 1.675 eV that randomly and abruptly switch their energies and intensities in excitation power below 200 μW (Figures S4d and S5b–e). Such a spectral wandering line can be a sign of defect-bound excitons.

There are also lines like 1.63 or 1.65 eV (Figure S4i,j) in which the intensity increases with power at a similar rate as the neutral exciton. (Likely due to strong nonradiative recombination channels, in the presented power range, it is not possible to fully saturate PL emission using powers only up to 270 μW.) These are also the lines visible in Figure 5 e, between 1.55 and 1.65 eV, during a scan in a magnetic field, which can be related to defect-bound or localized excitons.

As presented, WSe_2_ does not reveal a strain-affected lattice constant (Figure 2) or neutral/charged exciton energy shifted due to strain (Figure S3). More probable is the presence of defect-bound excitons, e.g., on selenium vacancies, which additionally explains why one can find sharp lines in similar energies in many different locations.

Last, the sharpest lines usually occur in places where hBN flakes are folded, cracked, or have their edges (Figures S4 and S5) and, therefore, are connected with the substrate’s morphology and most probably associated with the local strain field on the hBN substrate.^46^ The exception is the line at 1.645 eV (Figure S4g,h), which can be considered to be a dark, defect-localized exciton. To prove the randomness of the lines and show their significance in the spectrum, in Figure 5a, the average PL spectrum of 860 spots in the map is plotted.

Figure 5d displays the behavior of the PL response under an applied magnetic field measured in σ+ polarization. Besides the two main lines of X^0^ and CX, a branch of states at lower energies is also present, all redshifting in an increasing magnetic field. The Zeeman splitting energies of X^0^ and CX are also demonstrated in Figure 5e. Effective Lande *g* factors for X^0^ and CX are −4.4 and −2.4, respectively. While the *g* factor of the neutral exciton aligns with the expected values, the measured trion *g* factor is notably lower than the typical values reported for exfoliated WSe_2_.^47^ Due to the inability to spectrally resolve the singlet (SST) and triplet (TST) spin states of trions, the magnetic-field dependence of the oscillator strength becomes a critical factor. Assuming behavior analogous to that observed in WS_2_ (as reported in ref 48), the oscillator strength of the SST increases under σ+ polarization, while that of the TST decreases at a corresponding rate. This trend is reversed for σ– polarization.

If variation in the oscillator strength is taken into account, the effective trion *g* factor is expected to be approximately halfway, yielding a value around −2.2. This estimation is in good agreement with our experimental observations.

The literature reports show that, among epitaxial methods, CVD is mostly exploited for TMD growth. To compare with MBE-grown WSe_2_ on hBN, the results from Zhang et al.^27^ were chosen as representative of CVD, due to their leading film quality. Comparing the exciton and trion peaks in 80 K with our results, one can see that X^0^ fwhm’s are similar, 18.6 and 19.2 meV, while CX fwhm’s are noticeably smaller in our case (19.8 meV compared with 38.5 meV in ref 27). The authors of ref 27 claim the electron binding energy in trion to be ∼30 meV, while we can estimate it from our results to be close to 25 meV. Smaller binding energy can be attributed to low intrinsic doping or smaller WSe_2_ flakes, which can lead to shorter lifetimes of excitons.

Despite the fact that extensive progress in the MBE fabrication of WSe_2_ was achieved, there is plenty of room for further improvements. Among the necessary changes, we would like to place optimization of the process for continuous layer growth, suppressing multilayer formation. The WSe_2_ sample presented in this work had an hBN surface coverage of 55%. Above this value, we observe significant multilayer growth, and after exceeding a certain amount of material, excitonic lines are not resolved anymore and create a broad band (Figure S1). Therefore, actions toward a structurally uniform WSe_2_ layer inevitably expand applications of our material to transport studies or use in large-area heterostructures.

While in exfoliated materials, encapsulation is indispensable to the quality of optical measurements, our experiments show that covering as-grown WSe_2_ with hBN is not necessary and, as shown in Figure S2, does not further improve the results. The intensity of PL and the line widths stay the same, while the X^0^ and CX lines shift slightly toward lower energies.

Moreover, exfoliated hBN flakes are inherently size-limited; in order to achieve a functional size of the final product, we needed to find a source of large, uniform hBN layers. This is already partially achieved with MOCVD-grown hBN;^49^ however, while it can reach two inches of area, the atomic flatness of the surface is lacking, compromising the integrity of the MoSe_2_ layers grown atop. Nonetheless, the high applicational potential of hBN layers motivates investments in developing production methods of that material, and the results of these efforts are visible in many works,^50–55^ , giving hope that, in the near future, hBN produced in a bottom-up approach will be able to replace the exfoliated one.

We have showcased a three-step growth process of WSe_2_ by MBE on exfoliated hBN flakes, which provides a tool for the reproducible production of optically uniform monolayers. Through carefully chosen temperatures, durations, and W/Se flux ratios for each of the three steps, we have, achieved high optical effectiveness in WSe_2_ epitaxial films. We have accomplished an optical response comparable to that of exfoliated counterparts, without the need to fully encapsulate WSe_2_ with upper hBN. Crucially, we have developed WSe_2_ optical uniformity within hundreds of square micrometers, exhibiting high durability without capping. The samples preserve the optical properties for over 1 year if kept at moderately negative pressure, 0.1 Pa. Due to these improvements, for the first time, MBE-grown WSe_2_ has displayed clearly resolved neutral and charged exciton lines as well as localized and charged states. The behavior of those states was observed as a function of the external magnetic field and temperature. AFM images show a hexagonal structure of the layer to be free from major defects. With the use of TEM, we have proven growth in the 2H van der Waals regime. The integrity of the results presents a highly valuable advancement in the bottom-up production of 2D TMDs.

## Methods

### Substrate Preparation

The Si substrate (intrinsic semiconductor) with SiO_2_ of 90 nm was cleaved into smaller pieces and washed by using an ultrasonic cleaner in a three-step process. First, it was sonicated in isopropyl alcohol, then acetone, and finally demineralized water. After that, it was baked at 200 °C on the hot plate for 10 min. Directly after that, the substrates were stuck to tape with exfoliated hBN flakes and covered with a spare piece of foil to protect them from dust. Right before, the growth substrates were detached from the foil and placed in the MBE reactor.

### Molecular Beam Epitaxy (MBE)

The MBE reactor was from SVT Associates, Inc. The growth was conducted in the chamber dedicated to II–VI materials, under an ultrahigh vacuum with a background pressure below 1 × 10^–9^ Torr. A tungsten e-beam source and a selenium Knudsen cell were employed with source elements of tungsten in rod form (99.98% purity, 2 mm diameter, 35 mm length) and selenium granules (99.99999% purity). The MBE reactor was accompanied by reflection high electron energy diffraction (RHEED). The electron accelerating potential used was 8 kV.

### Ultrahigh-Vacuum (UHV) Atomic Force Microscopy (AFM)

High-resolution atomic force microscopy (HRAFM) measurements were performed in a vacuum chamber (∼5 × 10^–10^ mbar) with an Omicron VT XA microscope in contact mode by using NT-MDT CSG10 cantilevers. The results were processed using Scanning Probe Image Processor v.6.7.9 (Image Metrology A/S) software.

AFM images were obtained by using a Bruker Icon Dimensions microscope with a Nanoscope VI controller. Peak force tapping mode with a ScanAsyst-Air probe was employed.

### Transmission Electron Microscopy (TEM)

The sizes of the largest measured hBN flakes were around 40–60 μm. Cross sections were made by using a focused gallium ion beam with a HELIOS Nanolab 600 dual-beam electron–ion microscope. Lamellas were prepared in both directions: parallel to the flake edges and parallel to the cracks visible in the scanning electron microscopy (SEM) images.

Before the focused-ion-beam (FIB) process, an ∼10-nm-thick carbon layer was deposited on the flake surface to prevent charging during SEM imaging and the deposition of a protective platinum layer via e-beam deposition from a GIS platinum source. The standard method of cutting and removing the lamella using an Omniprobe nanomanipulator was applied.

During the thinning process to achieve electron transparency, an unusual problem occurred: the protective platinum curled, masking the thin part of the lamella. This issue is associated with the presence of metallic selenium above the WSe_2_ layer, which has poor mechanical strength compared to hBN and platinum and likely exhibits a chemical affinity for gallium.

As a result, the standard final thinning procedure was modified. Only small areas were thinned to a thickness suitable for high-resolution imaging (maximum up to 100 nm long), which prevented the protective platinum from curling but significantly reduced the useful observation area to just a few nanometers.

FIB cross-sectional imaging of the WSe_2_ layers was performed in aberration-corrected HR-TEM mode and HR-STEM mode using a HAADF detector, with a 76 mm camera length providing Z-contrast proportional to the average atomic number of the atomic columns. All TEM studies were conducted using an FEI Titan Cube 80-300 microscope operating at 300 kV.

### Optical Study

Photoluminescence (PL), reflection (REF), and photoluminescence excitation (PLE) measurements were performed in a helium-flow cryostat (Lakeshore Janis ST-500) at a temperature of 10 K. To reach a micrometer spatial resolution better than that of the exfoliated hBN flake sizes, a Nikon L Plan objective was used. Its magnification was 100× with NA = 0.7 and a working distance of 6.5 mm. The light source used during the reflectivity measurement was a halogen lamp filtered spatially via a pinhole and a set of lenses. The PLE source was a continuous-wave (CW) 532 nm semiconductor laser (Omicron LightHUB COBOLT Samba 100 mW), and the power at the sample position was kept close to 200 μW. Finally, to perform PLE measurements, a white-light supercontinuum laser (a SuperK EXTREME), together with a monochromator (a LLTF Contrast VIS HP8), provided an output light spectrum narrower than 2.5 nm (fwhm). To detect light coming back from the sample, an Andor SR-500i spectrometer was used with a diffraction grating of 600/500 nm and a Peltier-cooled CCD camera of Andor model DV420-FI. For Figures 5a–c, S4, and S5, the data were acquired 2 years later than those in the other figures, using the same sample. Therefore, the intensity of the signal might be slightly lower.

Determination of the excitonic Lande *g* factors was performed in a helium-bath cryostat in a gaseous helium atmosphere at 10 K in the PL configuration. The cryostat was an Oxford Spectromag 4000 with a superconducting magnet reaching a field of up to 10 T. Excitation was performed with a CW 532 nm semiconductor laser with well-defined linear polarization. The simultaneous detection in both σ+ and σ– polarization was achieved by using a quarter-waveplate and a Wollaston prism deflecting light along the spectrometer entrance slit. The spectrometer was SpectraPro HRS-300 (Princeton Instruments) with a grating of 600/500 nm and the CCD matrix camera iDus DU420A-OE (Andor). An aspherical, NA = 0.68, chromatic lens was used, optimizing the X^0^ and CX signal intensities.

## Supplementary Material



## Notes

Growth details and power- and temperature-dependent data (PDF)
