# Correction to “WSe_2_ Monolayers Grown by Molecular Beam Epitaxy on hBN”

**DOI:** 10.1021/acs.nanolett.6c01257

**Published:** 2026-04-20

**Authors:** Julia Kucharek, Mateusz Raczyński, Rafał Bożek, Anna Kaleta, Bogusława Kurowska, Marta Bilska, Sławomir Kret, Takashi Taniguchi, Kenji Watanabe, Piotr Kossacki, Mateusz Goryca, Wojciech Pacuski

In the original publication, one of the grant numbers is incorrect. This sentence in the Acknowledgments section should read as “We acknowledge financial support from the National Science Centre, Poland (Project Nos. 2020/39/B/ST3/03251, 2021/41/B/ST3/0418**3**, and 2023/49/N/ST11/04125).”

