# Supplementary material for: WSe2 Monolayers Grown by Molecular Beam Epitaxy on hBN
Source: Nano Lett. 2025 Dec 7;25(50):17275–84. doi: 10.1021/acs.nanolett.5c04228 (PMC12715837; doi:10.1021/acs.nanolett.5c04228)
Supplement: Supplementary file 1 [file nl5c04228_si_001.pdf]

SUPPLEMENTARY INFORMATION

WSe_2 Monolayers Grown by Molecular Beam Epitaxy on hBN

Julia Kucharek*[1], Mateusz Raczyński[1], Rafał Bożek[1], Anna Kaleta[2], Bogumiła Kurowska[2], Marta Bilska[2], Sławomir Kret[2], Takashi Taniguchi[3], Kenji Watanabe[4], Piotr Kossacki[1], Mateusz Goryca[1], Wojciech Pacuski[1]

[1] Institute of Experimental Physics, Faculty of Physics, University of Warsaw, Pasteura 5, 02-093 Warsaw, Poland,

[2] Institute of Physics, Polish Academy of Sciences, Aleja Lotników 32/46, 02-668 Warsaw, Poland,

[3] Research Center for Materials Nanoarchitectonics, National Institute for Materials Science, 1-1 Namiki, Tsukuba 305-0044, Japan

[4] Research Center for Electronic and Optical Materials, National Institute for Materials Science, 1-1 Namiki, Tsukuba 305-0044, Japan

Sample	Growth temperature [°C]	Growth time [h]	Annealing temperature [°C]	Annealing time [h]
UW2144	300	2.5	800	2
UW2145	300	3	800	2

Table S1. Growth details of samples UW2144 and UW2145 investigated in the main text: growth temperature and time, annealing temperature and time.

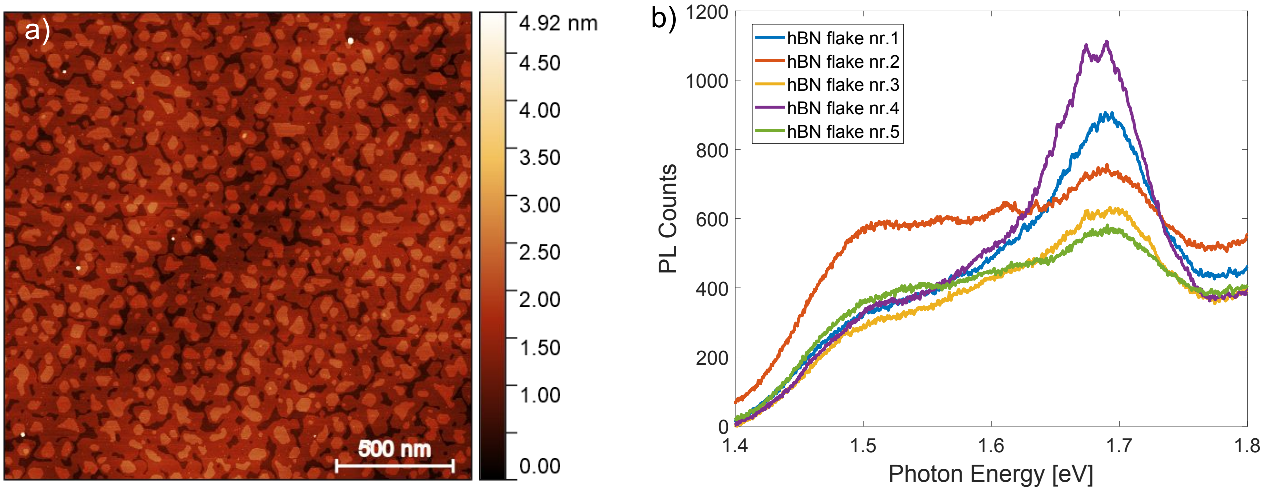

Figure S1. a) AFM image of sample (UW2083) exceeding 70% of hBN surface coverage. Monolayer plane is nearly coalesced and significant part of the film is composed of bilayer. b) PL spectra for a) measured in four different places on the sample. All shows that PL signal in the region of neutral and charged excitons reveals one broad band with a maximum ~1.69 eV, below charged exciton energy. Observed emission energy is higher than the one from bilayer noticed in literature, but it is too low to be created directly from connection of exciton and trion lines. 515 nm laser with power of 800 μW was used. Measurement temperature was 10 K.

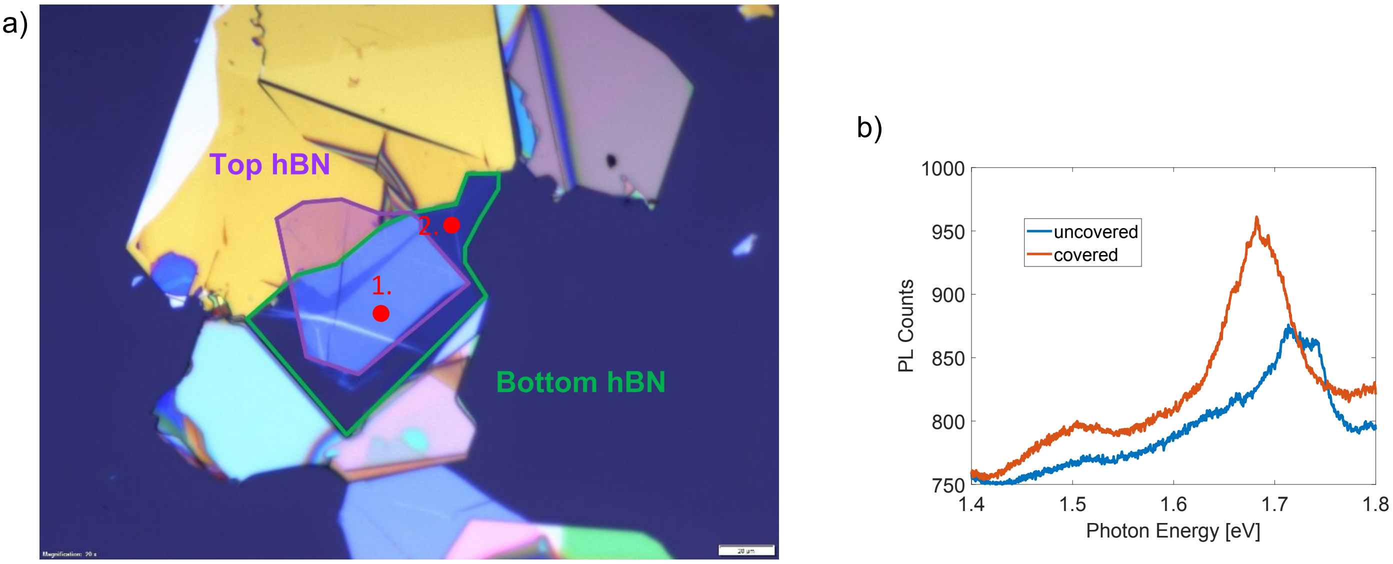

Figure S2. a) Optical image of WSe_2 film grown by MBE on hBN (UW2266) covered with top hBN through polymer stamp dry method. After the transfer, the sample was re-annealed using the same procedure as for post-growth annealing. Spots 1. (uncovered) and 2. (covered) were measured for comparison after finishing the entire process. b) PL spectra of covered (orange) and uncovered (blue) regions. 532 nm laser with power of 600 µW was used. Measurement temperature was 10 K.

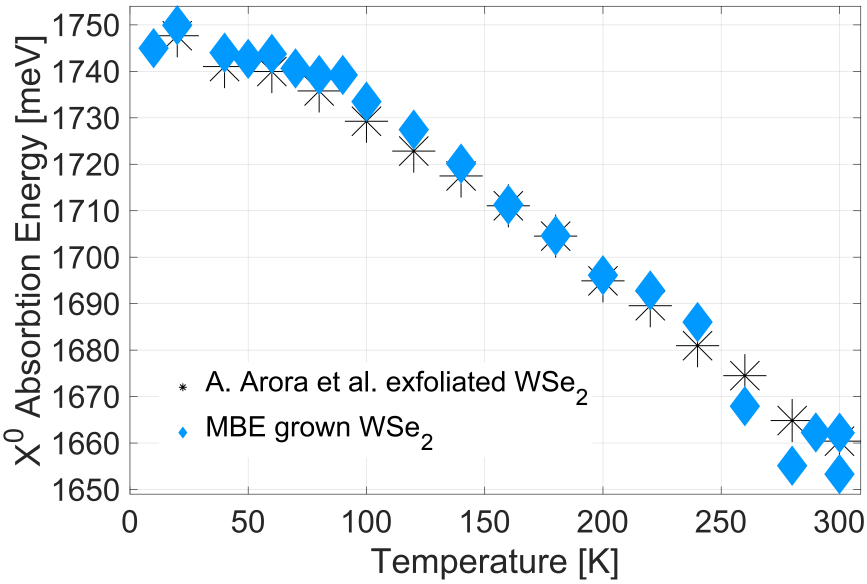

Figure S3. Comparison of X^0 evolution in temperature. Black stars are points measured by A. Aurora et al. [1] on exfoliated WSe_2 encapsulated in hBN, and blue diamonds are results from our MBE-grown WSe_2. No significant offset in energies is visible, which suggest the strain in both types of samples is similar, close to zero.

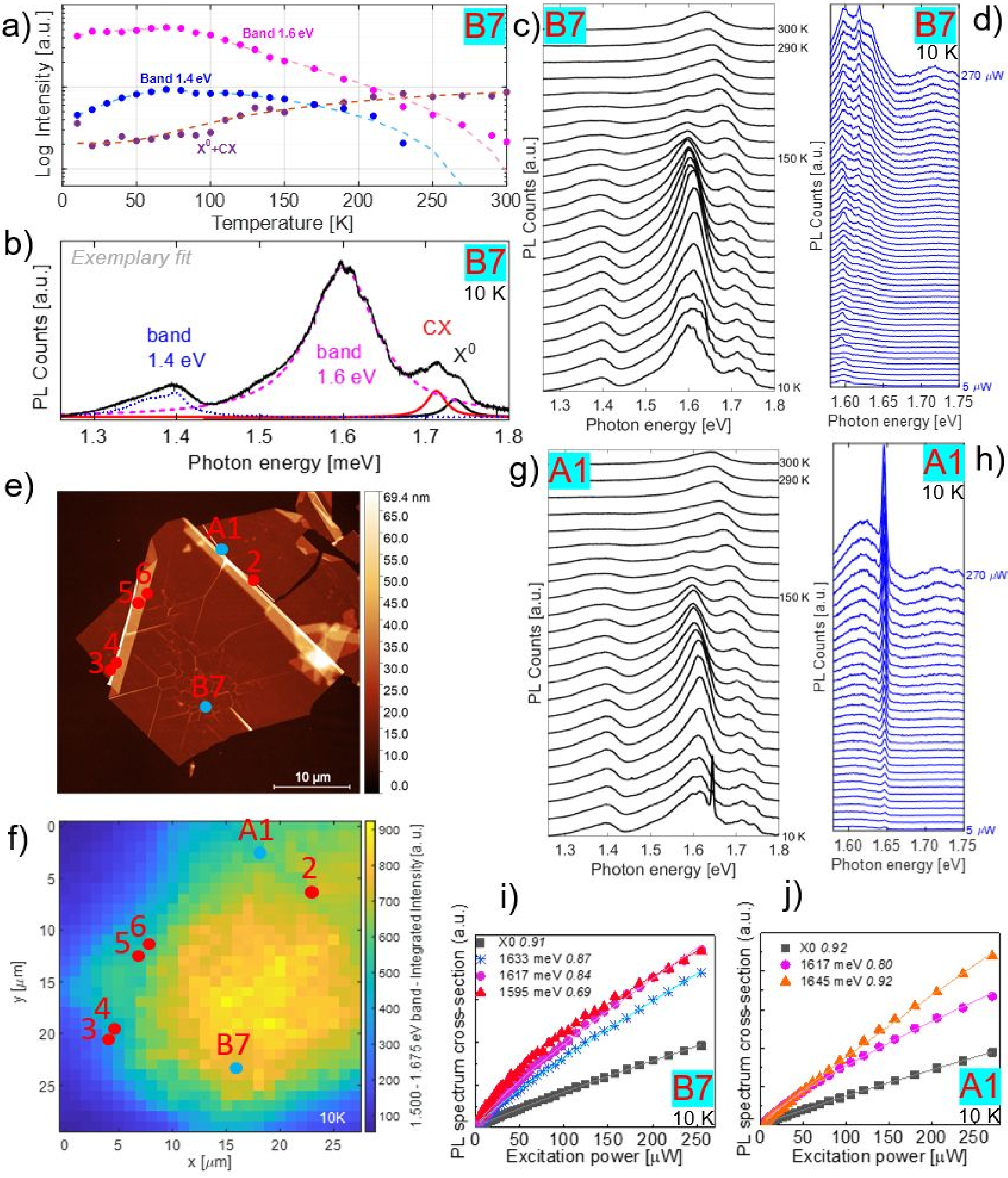

a)
B7
Band 1.6 eV
Band 1.4 eV
X0+CX
Log Intensity [a.u.]
Temperature [K]
b)
Exemplary fit
B7
10 K
band
1.4 eV
band
1.6 eV
CX
X0
PL Counts [a.u.]
Photon energy [meV]
c)
B7
300 K
290 K
150 K
10 K
PL Counts [a.u.]
Photon energy [eV]
d)
B7
10 K
270 μW
5 μW
PL Counts [a.u.]
Photon energy [eV]
e)
A1
2
6
5
4
3
B7
10 μm
69.4 nm
f)
A1
2
6
5
4
3
B7
10K
y [μm]
x [μm]
1.500 - 1.675 eV band - Integrated Intensity [a.u.]
g)
A1
300 K
290 K
150 K
10 K
PL Counts [a.u.]
Photon energy [eV]
h)
A1
10 K
270 μW
5 μW
PL Counts [a.u.]
Photon energy [eV]
i)
X0 0.91
1633 meV 0.87
1617 meV 0.84
1595 meV 0.69
B7
10 K
PL spectrum cross-section (a.u.)
Excitation power [μW]
j)
X0 0.92
1617 meV 0.80
1645 meV 0.92
A1
10 K
PL spectrum cross-section (a.u.)
Excitation power [μW]

Figure S4. Temperature (black waterfalls) and power-dependent (blue waterfalls) study of the WSe_2 monolayers grown on hBN flake, shown in e). a) Temperature dependence of PL intensity measured in point B7. There are 3 independent plots: violet – summed neutral and charged excitons intensities, pink – band centred at 1.6 eV, and blue dedicated to the band at 1.4 eV. For all the datasets, the activation/deactivation function (1-2) was fitted, and the activation/deactivation energies were found (Table S1). It is worth noting that the pink curve reaches the maximum at 70 K and from 10 K to 70 K its intensity rises about 26%, while for the blue one maximum is at 80 K with an increase of ~108%. b) Exemplary spectrum from c) with fitted Lorentzian curves to illustrate how points in a) were obtained. c) Temperature dependence measured in point B7. For all of the temperature-dependent data in figures S4, points from 10 K to 150 K are acquired every 10 K; points from 170 K to 290 K every 20 K; the last data point is at 300 K. d) Power-dependence measured in point B7. For all of the power-dependent data in Figure S4, 50 evenly distributed data points from 5 µW to 270 µW were tested. e) AFM picture of the measured flake. Red labelled points were measured in changing excitation power. Additionally, two of them marked in blue were also measured in changing temperature. f) PL map of the part of the flake in e). Labelling of the data-points stays the same as in e). g) Temperature dependence measured in point A1. h) Power-dependence measured in point A1. i) PL intensity in function of excitation-power for cross-section of approximate width of 8 meV; presented lines: d) X0, 1633 meV, 1617 meV, 1595 meV with fitted exponent x in equation (3) in italic j) PL intensity in function of excitation-power for 4 lines presented also in h): X0, 1645 meV, 1617 meV with fitted exponent x in equation (3) in italic.

	X^0 + CX	Band 1.6 eV	Band 1.4 eV
Activation energy	18. 42 meV	2.72 meV	4.23 meV
Deactivation energy	-	0.78 meV	39.42 meV

Table S1. Values of activation/deactivation energy calculated from Fig. S4a.

Arrhenius equations:

$$I = I_0 + I_A e^{-\frac{Ea}{kT}} \quad (1)$$

$$I = I_0 + I_D\left(1 - e^{-\frac{Ea}{kT}}\right) \quad (2)$$

Power dependance:

$$I \propto P^x \quad (3)$$

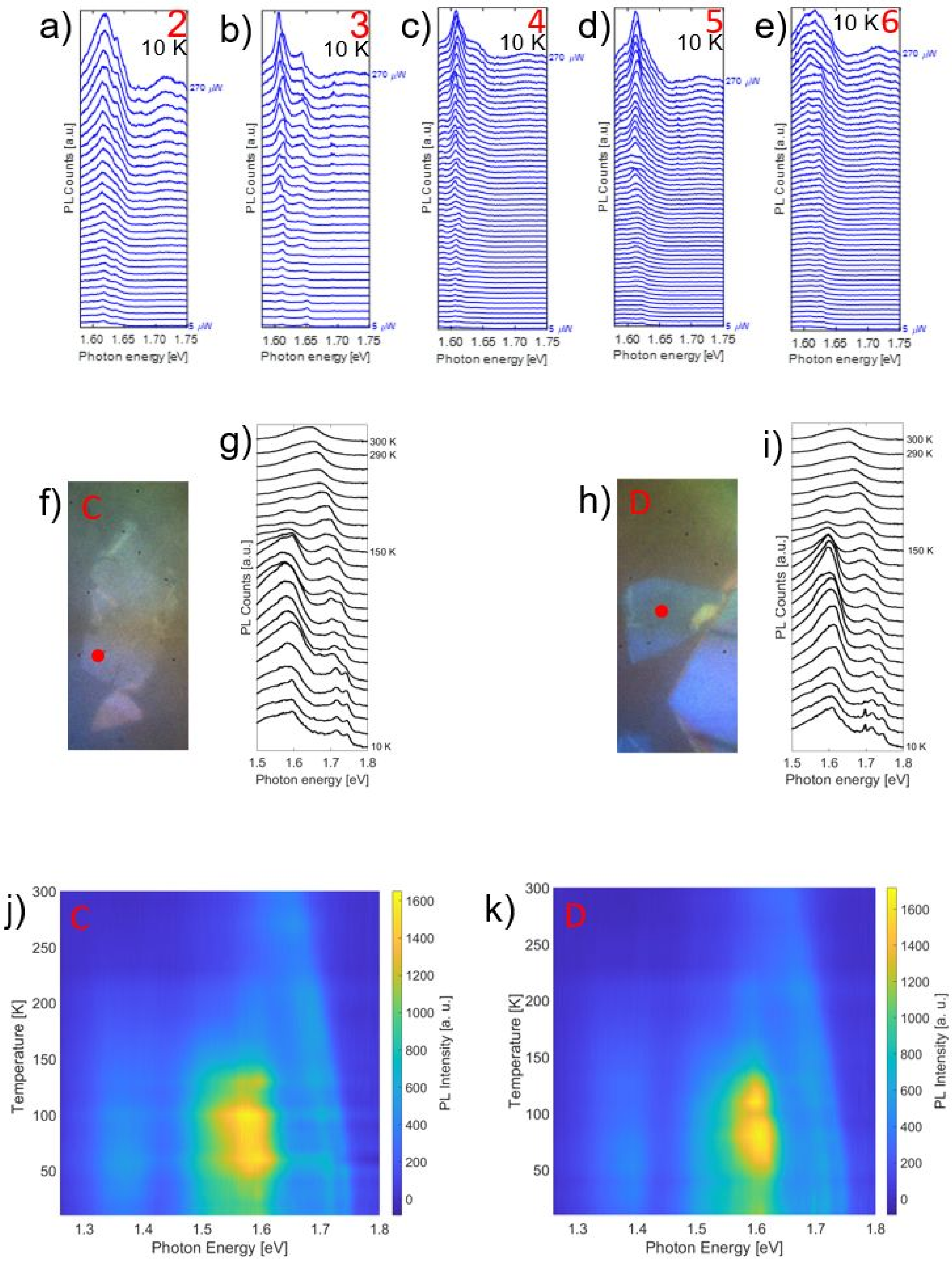

a)
2
10 K
b)
3
10 K
c)
4
10 K
d)
5
10 K
e)
10 K
6
270 μW
5 μW
PL Counts [a.u]
1.60 1.65 1.70 1.75
Photon energy [eV]
f)
C
g)
300 K
290 K
150 K
10 K
PL Counts [a.u.]
1.5 1.6 1.7 1.8
Photon energy [eV]
h)
D
i)
300 K
290 K
150 K
10 K
PL Counts [a.u.]
1.5 1.6 1.7 1.8
Photon energy [eV]
j)
C
Temperature [K]
300 250 200 150 100 50
1.3 1.4 1.5 1.6 1.7 1.8
Photon Energy [eV]
PL Intensity [a. u.]
1600 1400 1200 1000 800 600 400 200 0
k)
D
Temperature [K]
300 250 200 150 100 50
1.3 1.4 1.5 1.6 1.7 1.8
Photon Energy [eV]
PL Intensity [a. u.]
1600 1400 1200 1000 800 600 400 200 0

Figure S5. a)-e) Power-dependence measured in points 1-6. For all of the power-dependent data in figure S4 50 evenly distributed data points from 5 µW to 270 µW were tested. f) Optical image of flake C measured in the place of red dot. g) Temperature-dependent data measured on flake C. For all of the temperature-dependent data in figures S5 points from 10 K to 150 K are acquired every 10 K; points from 170 K to 290 K every 20 K; the last data point is at 300 K. h) Optical image of flake D measured in the place of red dot. i) Temperature-dependent data measured on flake D. j) Temperature-dependent data measured on flake C in a form of a colour map. k) Temperature-dependent data measured on flake D in a form of a colour map.

Figure S6. The following sets of AFM pictures compared with PL spectra are illustration for growth parameters choice. All the PL spectra were measured in 10 K.

Growth parameters:

- Growth temperature: A temperature of 300°C provides a balance between W/Se edges and reduces the number of nucleation centres. Material grown at 500ºC and 400ºC doesn't reveal PL signal.

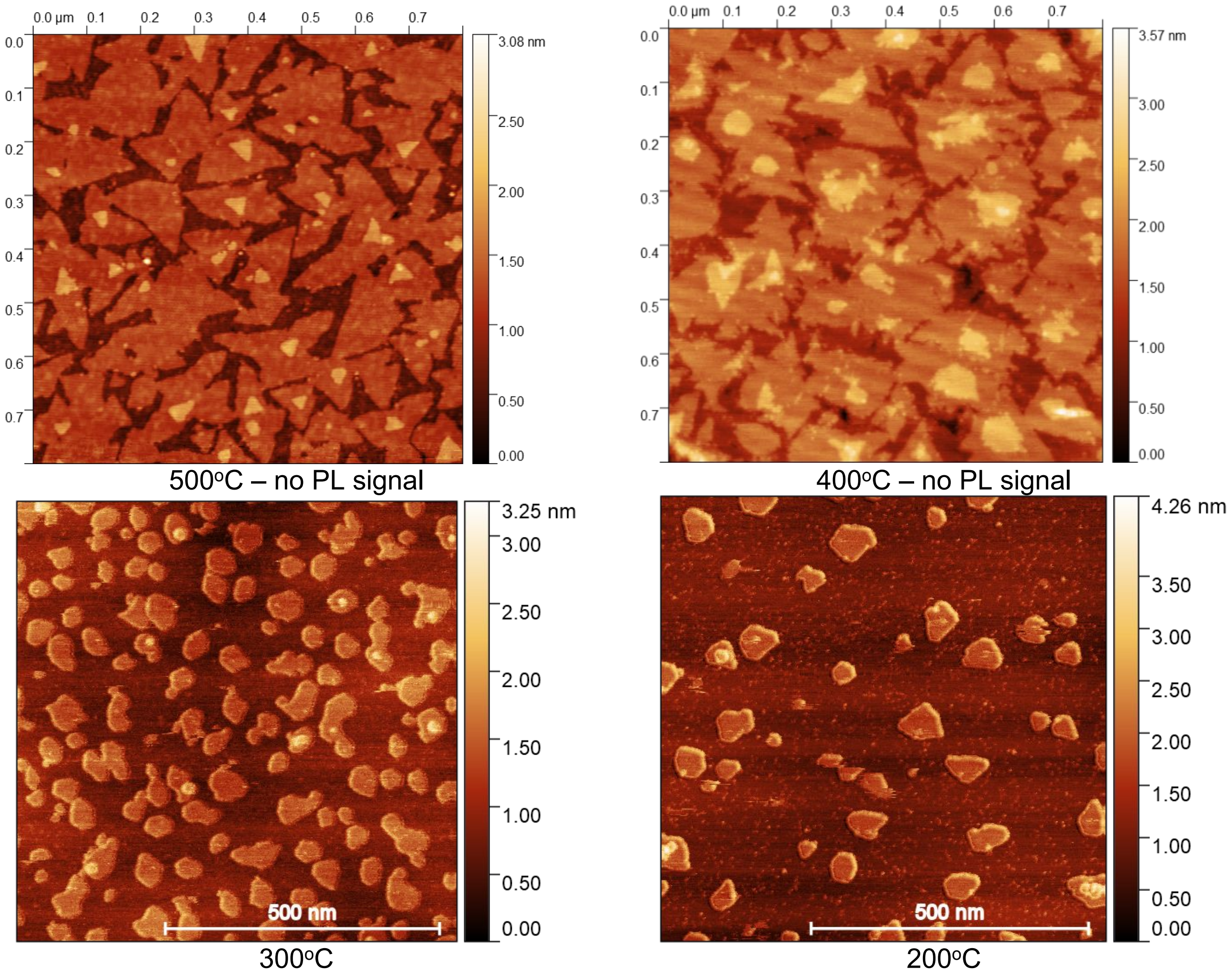

- Coverage: by maintaining coverage at ~6o%, the growth of multilayers is reduced. There is enough material for PL observation, which is not yet affected by multilayer peaks.

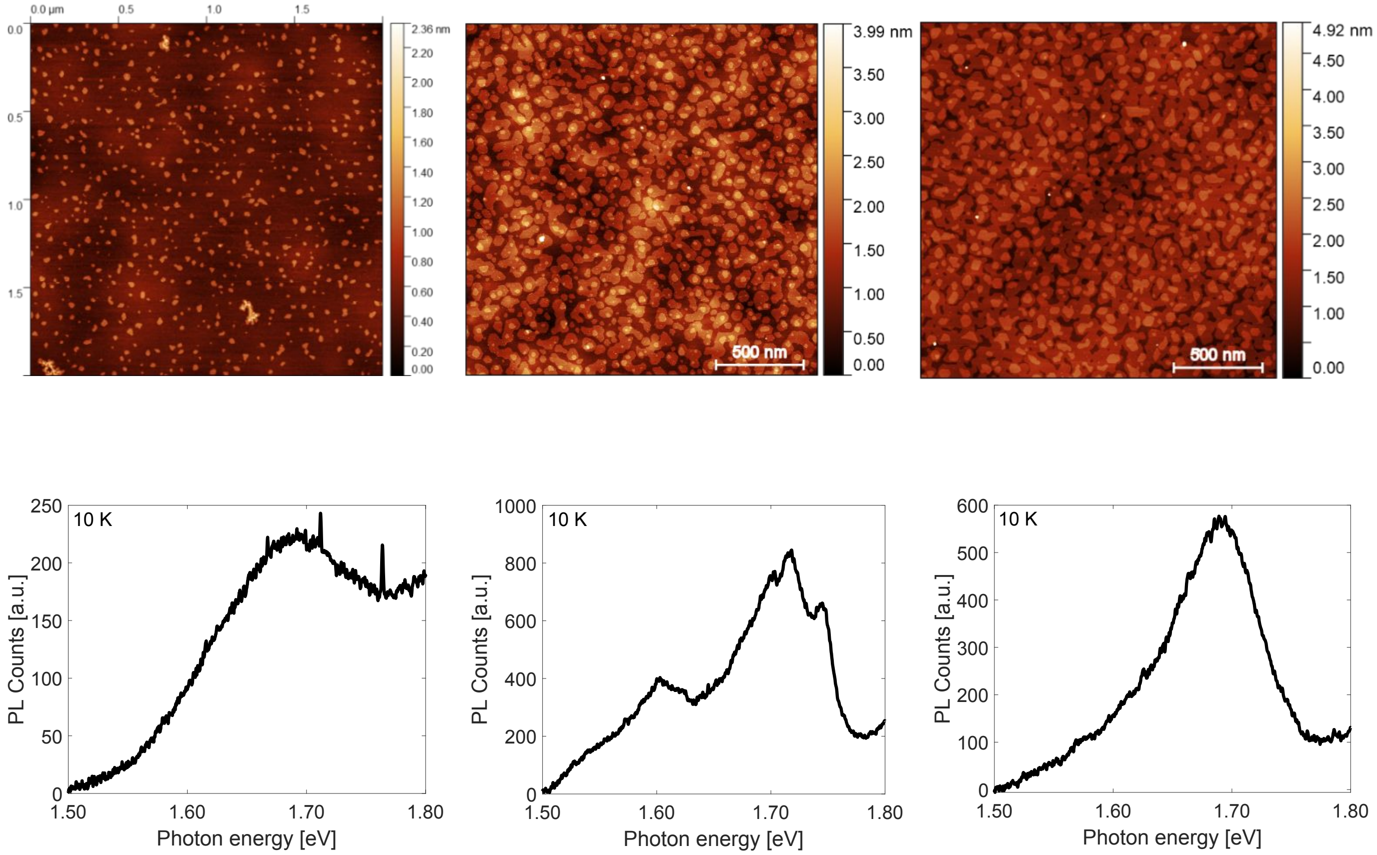

<30% ~60% >80%

- Post-growth annealing temperature: Temperature of 800°C reduces 3D growth, sharpens flakes' edges, and improves PL intensity.

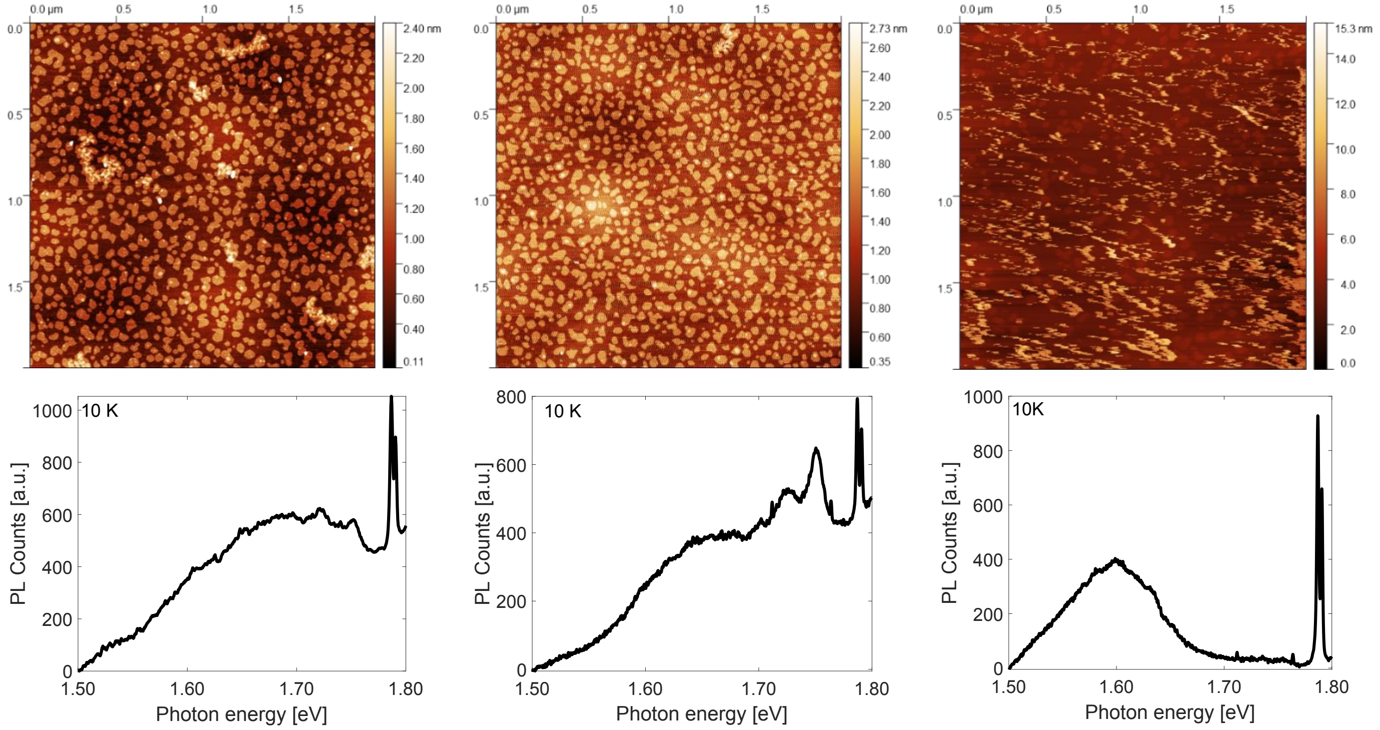

750°C 800°C 850°C

- Pre-growth annealing: removes strain and bubbles from hBN flakes, facilitating uniform growth.

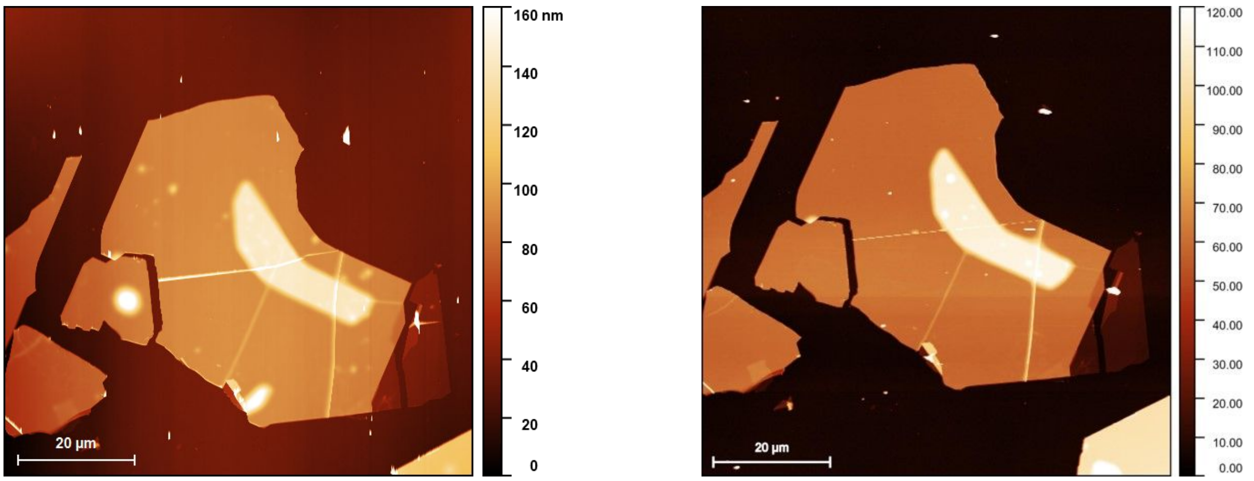

none 1 h in 700°C

- Selenium flux: is **crucial** for sharp, hexagonal flakes and a strong PL signal.

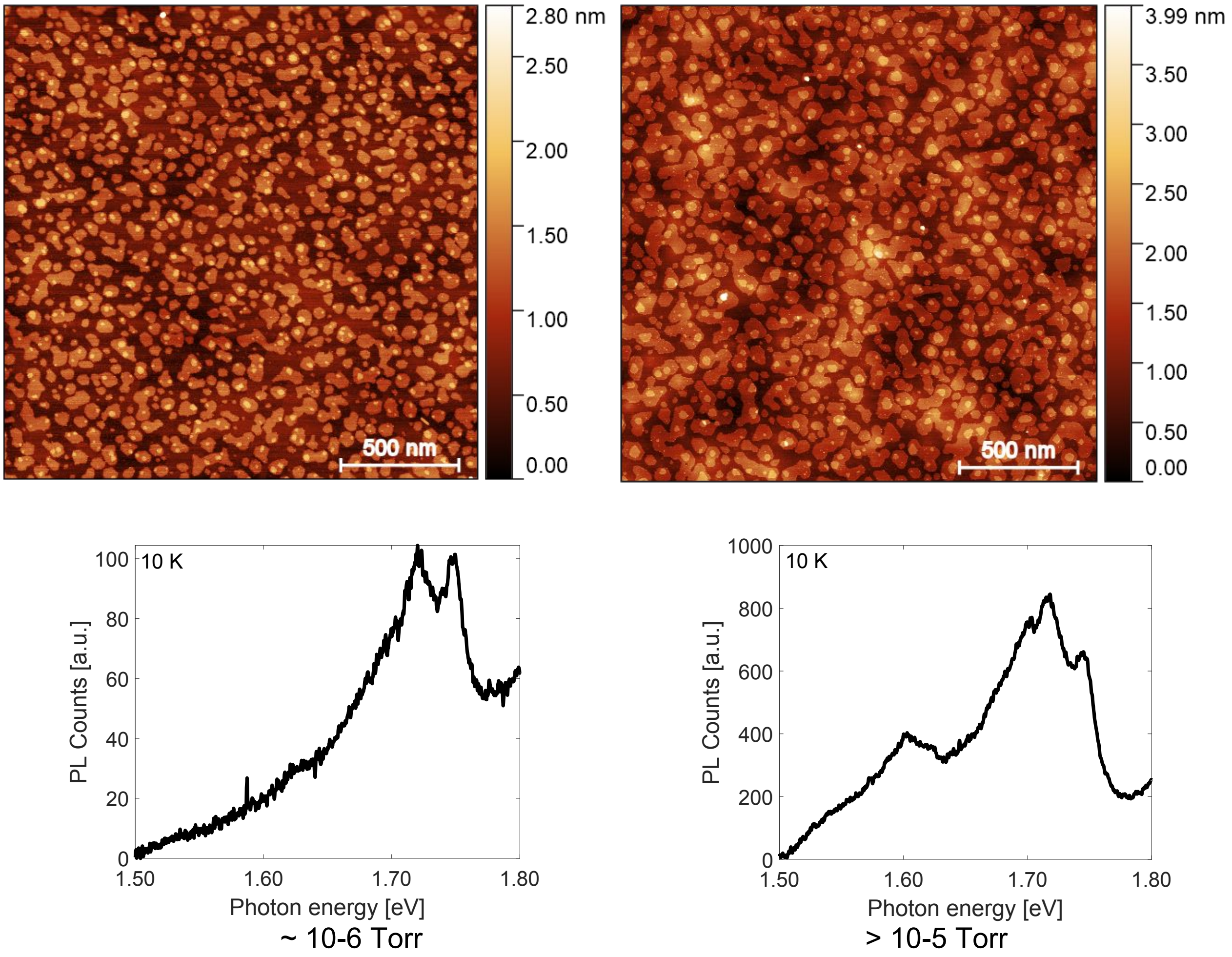

References

(1) Arora, A.; Koperski, M.; Nogajewski, K.; Marcus, J.; Faugeras, C.; Potemski, M. Excitonic resonances in thin films of WSe2: from monolayer to bulk material. *Nanoscale* **2015**, *7* (23), 10421–10429. DOI: 10.1039/c5nr01536g.
